# Depth Control of an Underwater Sensor Platform: Comparison between Variable Buoyancy and Propeller Actuated Devices

**DOI:** 10.3390/s24103050

**Published:** 2024-05-11

**Authors:** João Falcão Carneiro, João Bravo Pinto, Fernando Gomes de Almeida, Nuno A. Cruz

**Affiliations:** 1 Instituto de Ciência e Inovação em Engenharia Mecânica e Engenharia Industrial, Faculdade de Engenharia, Universidade do Porto, Rua Dr. Roberto Frias, s/n, 4200-465 Porto, Portugal; fga@fe.up.pt; 2 Faculdade de Engenharia, Universidade do Porto, Rua Dr. Roberto Frias, 400, 4200-465 Porto, Portugal; jpp.professional@gmail.com (J.B.P.); nacruz@fe.up.pt (N.A.C.); 3 INESC TEC, Faculdade de Engenharia, Universidade do Porto, Rua Dr. Roberto Frias, 4200-465 Porto, Portugal

**Keywords:** underwater sensing, autonomous underwater vehicles, variable buoyancy, energy savings, depth control

## Abstract

Underwater long-endurance platforms are crucial for continuous oceanic observation, allowing for sustained data collection from a multitude of sensors deployed across diverse underwater environments. They extend mission durations, reduce maintenance needs, and significantly improve the efficiency and cost-effectiveness of oceanographic research endeavors. This paper investigates the closed-loop depth control of actuation systems employed in underwater vehicles, focusing on the energy consumption of two different mechanisms: variable buoyancy and propeller actuated devices. Using a prototype previously developed by the authors, this paper presents a detailed model of the vehicle using both actuation solutions. The proposed model, although being a linear-based one, accounts for several nonlinearities that are present such as saturations, sensor quantization, and the actuator brake model. Also, it allows a simple estimation of the energy consumption of both actuation solutions. Based on the developed models, this study then explores the intricate interplay between energy consumption and control accuracy. To this end, several PID-based controllers are developed and tested in simulation. These controllers are used to evaluate the dynamic response and power requirements of variable buoyancy systems and propeller actuated devices under various operational conditions. Our findings contribute to the optimization of closed-loop depth control strategies, offering insights into the trade-offs between energy efficiency and system effectiveness in diverse underwater applications.

## 1. Introduction

Understanding of the marine environment is becoming increasingly important across several domains, including oceanic energy [1], ecosystems [2], raw materials [3], and understanding the ocean’s influence on climate [4], weather patterns, etc. [5]. Consequently, monitoring different ocean parameters holds significant importance, prompting endeavors in academia and industry to develop devices that are cost-effective and robust and require minimal human intervention [6]. In the design of ocean monitoring devices, achieving energetic autonomy is a key focus, as it not only dictates mission types and durations but also influences the range of sensors and measurements that can be employed, with enhanced energy availability enabling broader capabilities [5,7]. 

In this context, underwater gliders [8] emerge as notable and successful solutions, capable of completing missions spanning months without maintenance or support. The basic mechanism behind the low energetic consumption of gliders lies in its Variable Buoyancy Module (VBM), which allows travelling at the expense of low energy buoyancy changes. The utilization of variable flotation engines is also central to well-established solutions like the Argo floats [9]. In this growing energy demand scenario, the use of VBM as auxiliary engines for thruster-powered devices becomes increasingly appealing, leading to the so-called hybrid AUVs [10]. In this approach, the VBM may be used to provide neutral buoyancy for vertical displacements while the propellers handle motion in other directions. One relevant scenario is the operation in estuaries, where the changes in water density cause large variations in the vehicle buoyancy. This process is the reverse of the one in underwater gliders, where hybrid gliders now include a thruster to act whenever high currents or tight maneuvers are required [11]. Beyond energy constraints, the use of VBM for depth control holds great importance for specific missions that require a minimal acoustic signature, such as military missions [6] or marine observation to avoid disturbing studied species [12]. However, despite this significance, few studies in the literature have focused on determining the circumstances favoring VBM use over propellers. The authors have previously contributed to this area by developing static models, allowing the calculation of the power consumed by a VBM, both in electric and hydraulic solutions [13], as well as dynamic models based on experimental data for electrical actuated VBMs [14]. 

Nevertheless, the comparative energy consumption of propeller and VBM solutions under closed-loop control remains largely unexplored in the literature. Specifically, the development of control laws targeting low energy consumption and the trade-offs involved in balancing energy consumption and depth control performance require further investigation. Some studies can be found on depth control, like, for instance, refs. [15,16]. In [15], three depth controllers are developed and tested for a profiling float specifically designed for monitoring thermoclines. The float uses a linear electric actuator to achieve buoyancy change. The proposed segment PD control method is based on switching a velocity PD controller to a depth PD controller when a given error band is reached. Simulation and experimental results show that a maximum depth error of around 0.3 m is achieved, for depth steps up to 60 meters. However, no insights are given regarding the absolute or relative energy consumption of each control method. In [16], a combinational controller including a PID controller, linear quadratic regulator (LQR), and sliding mode controller (SMC) is designed for the depth control of a bellows-like electrically driven VBM. It is shown, through simulations and experiments for several depth steps, that the proposed combinational controller outperforms all individual ones regarding control performance measured by rise time, overshoot, and settling time. However, once again, there is no remark regarding the energy spent by each controller or by the proposed combinational one. The only study the authors could find presenting a quantitative comparison between energy consumption of two VBM controllers was [17]. In this study, a linear quadratic regulator (LQR) is compared against a two-stage cascaded proportional-derivative controller (2S-PD) using depth and vertical velocity in the feedback loops. It is shown that the LQR with a wide deadband in both depth and vertical velocity can significantly reduce the energy consumption in comparison to the 2S-PD controller. However, no deadband was implemented in the 2S-PD controller, so a full comprehension of the benefits of the LQR controller is not possible. Also, results do not consider any buoyancy disturbance, so the robustness of the controllers is not clear.

As far as the authors could ascertain, energy consumption figures are primarily available in studies combining propellers and VBMs, within hybrid control strategies. For instance, study [11] introduced a Slocum hybrid glider equipped with folding propeller blades to reduce drag during buoyancy-driven flight. The study highlights several advantages of this hybrid approach, including enhanced efficiency in horizontal flight due to neutral buoyancy at depth, improved performance in overcoming strong currents and surfacing in low-density waters, and the ability to optimize the efficiency of the pump-driven VBM by employing the propeller when necessary. However, the study lacks specific energy gain figures and detailed insights into control strategy benefits. In another study [7], a vehicle incorporating both propellers and a hydraulic VBM was explored, testing four control strategies: propeller-only, VBM-only, and two hybrid approaches. The hybrid strategies involved (i) sequential control, where the propellers initially stabilize the depth followed by transferring control to the VBM, and (ii) simultaneous control using both actuation systems with different controllers. While the study indicates some advantages of hybrid strategies, uncertainties remain regarding the generalizability of results to different depth steps and the absence of experiments including VBM deactivation or buoyancy disturbances. Bi et al. [18] proposed a hybrid depth control strategy integrating an on–off hydraulic VBM for swift depth adjustments and fin control for energy conservation during cruising with propellers. The strategy enables efficient diving and surfacing without propeller usage and effective hovering control with low power consumption. However, the study lacks energy consumption estimates or measurements, studies on robustness to external disturbances, and a detailed description of the propeller control strategy.

Overall, existing studies suggest potential gains in energy efficiency with appropriate depth control strategies, but a systematic approach is lacking. Further research is needed to quantify energy efficiency, address control robustness, and evaluate performance under various environmental conditions. This work contributes to this endeavor by providing tools for systematic comparison between propeller- and VBM-driven depth controllers, aiming to address key questions surrounding their usage and performance trade-offs: When using a VBM, should one use one single controller for depth control or two cascaded ones, one for depth and another for volume control? Should deadbands be used in those controllers? If so, for depth control, for volume control, or for both? How can (internal and external) buoyancy disturbances be counteracted? What is the trade-off between depth control performance and energy consumption for a given control law?

The contributions of this work include (i) the development of a linearized model, accounting for real-world nonlinearities, of depth motion for a propeller or VBM actuated sensor platform; (ii) the development and simulation testing of several PID-based controller structures for this platform, including the robustness to buoyancy disturbances; (iii) examples illustrating the trade-offs between depth control performance and energy consumption for the developed controllers.

This work is organized as follows: Section 2 presents the prototype previously developed by the authors. After a short description of the prototype modules in Section 2.1, the several partial models are presented in Section 2.2, Section 2.3 and Section 2.4. The parameters of the presented models are determined in Section 2.5. Section 3 presents in detail the controllers proposed in this work and Section 4 presents the simulation results when the controllers developed in Section 3 are applied to the models developed in Section 2 in different operational scenarios. Finally, Section 5 draws the main conclusions obtained in this work.

## 2. Prototype Description and Model

### 2.1. Prototype Description

**Figure 1:**
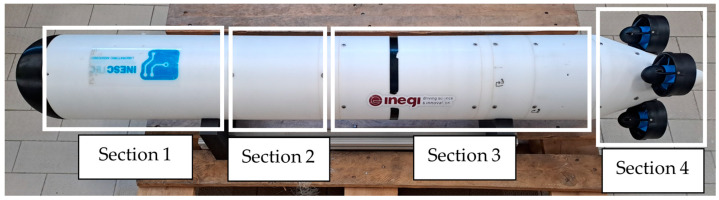
A picture of the updated prototype.

To explore potential advantages of a buoyancy-driven device on energy consumption, the authors developed a prototype with a VBM as documented in several previous works, like, for instance, in [19]. The VBM is designed for integration into small-sized AUVs based on modular building blocks or to be independently used as a buoy for vertical profiling missions. The prototype underwent enhancements, as described in [14], which introduced a Main Control Unit (MCU), for autonomous operation. Within the MCU, there is an Arduino Uno and a Turnigy high capacity 14.8 V Cell Battery to supply power to the whole prototype. In this setup, a Gravity IC Digital Wattmeter was included to measure the power consumption of the prototype actuating devices. This sensor provides readings for (i) current with a range of 0 to ±8 A with a 1 mA resolution and ±0.2% full-scale relative error, (ii) voltage with a range of 0 to 26 V with a 4 mV resolution and ±0.2% relative error, and (iii) power with a range of 0 to 206 W with a 20 mW resolution. Building on these enhancements, the present study incorporated a Propeller Module (PM) into the prototype, facilitating energy consumption comparison. Depicted in Figure 1, the prototype is composed of four main sections: Section 1 is the MCU, Section 2 is an intermediate section for floatation foam and Sections 3 and 4 are the VBM and the PM, respectively. Its full length is 1616 mm with an outer radius of 200 mm and its dry weight is 36 kg. Buoyancy change is achieved by pumping seawater via a diaphragm-sealed piston mechanism. The piston is driven by an electrical motor coupled to a mechanical transmission and a spindle. This VBM allows a total volume change of approximately ${\pm D}_{t}=\pm 350$ cm^3^ and can operate up to 100 m. Further details of the VBM can be found in [14].

**Figure 2:**
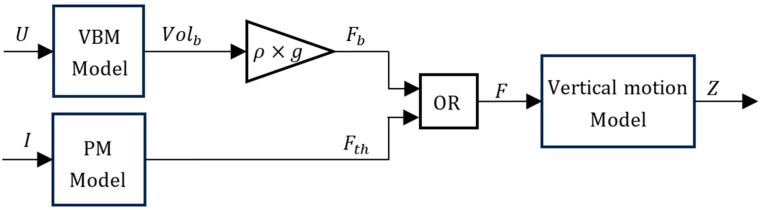
Prototype model.

The main focus of this work is to compare the energy consumption between the VBM and the PM without having to rely on real-world tests but instead on simulations or simple lab experiments. To this end, a detailed model of the prototype is required. The vertical motion of the prototype is achieved by either changing its buoyancy with the VBM or using the propeller thrust. In [19], the model of the VBM actuated prototype was presented. The updated version of the prototype can be described by the model in Figure 2, where it is assumed that the weight of the vehicle is perfectly counterbalanced by the buoyancy force caused by its fixed volume. The fixed volume is the volume that the vehicle has when the movable piston is at its middle point.

In Figure 2, U and *I* are the control actions to either the VBM or the PM, respectively; Z is the prototype depth (which increases with increasing depths); and F is the force responsible for driving the prototype vertical motion (positive F increases buoyancy, leading to decreasing depth). The force F can be either $F_{th}$, the propeller thrust, or $F_{b}$, the variable buoyancy force. ${Vol}_{b}$ is the variable buoyancy volume of the prototype, which generates the variable buoyancy force $F_{b}$. External disturbances are accounted for in the vertical motion model as will be described in the next section.

### 2.2. Vertical Motion Model

**Figure 3:**
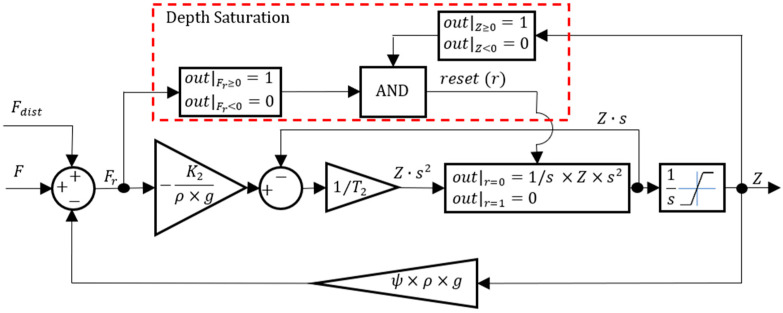
The vertical motion model of the prototype.

Based on the work developed in [19], the vertical motion model of the prototype is presented in the block diagram of Figure 3.

In Figure 3, $K_{2}$ and $T_{2}$ are the depth dynamics parameters identified in [19]. ρ is the water mass per unit volume, g is the acceleration of gravity, and ψ is a parameter expressing the loss of volume per meter depth due to the variation of volume of the prototype structure. $F_{dist}$ is a disturbance force acting on the vehicle, such as a water density variation or an error during the neutral buoyancy trimming of the prototype, and $F_{r}$ is the resultant force acting on the prototype. The model developed in [19] is valid when the prototype is completely submerged. However, in many practical situations, the prototype is floating at the water surface level. For this reason, the depth Z is saturated so that it is never negative. When Z is saturated and $F_{r}$ is negative, the integral of the prototype acceleration is reset to zero, so that only when the direction of $F_{r}$ is away from the surface can the integration of the acceleration start.

### 2.3. VBM Model

**Figure 4:**
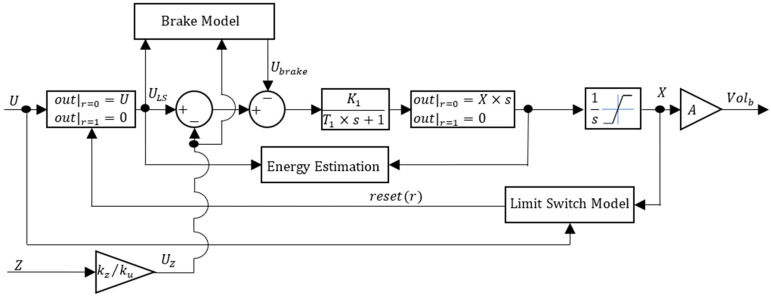
VBM nonlinear model.

In [14], a linearized model of the VBM was presented. In this study, we expand the model developed in [14] by incorporating several real-world nonlinearities. The complete nonlinear version is presented in Figure 4.

**Figure 5:**
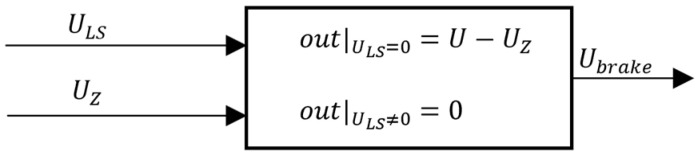
Brake model.

**Figure 6:**
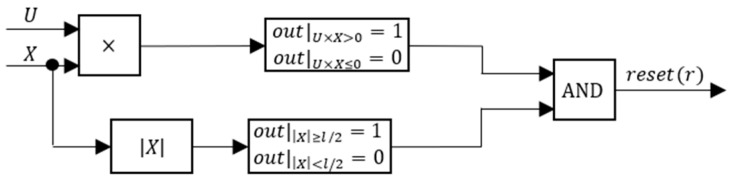
Limit switch model.

The parameters $K_{1}$ and $T_{1}$ are the actuator velocity dynamics steady-state gain and time constant, respectively, that were experimentally identified in [14]. The pressure due to depth Z directly affects the torque required for actuator motion. This effect is expressed through the equivalent depth voltage $U_{Z}$, the product between Z and the ratio $k_{z}/k_{u}$. The constant $k_{z}$ accounts for the transmission ratio between the force exerted by the outside pressure and the corresponding torque caused on the motor, while $k_{u}$ relates the applied voltage with the stall torque. The linear actuator includes a mechanical brake to prevent back-driving when there is no power. $U_{break}$ is the equivalent brake voltage detailed in the brake model presented in Figure 5. Note that the actuator piston position X is bounded by its stroke, hence the limited integrator. When the actuator reaches either stroke limit, the limit switches are triggered and the reset signal *r* rises to 1, making the control action become zero. The limit switch model is presented in Figure 6. The VBM buoyancy volume ${Vol}_{b}$ is the limited position X multiplied by the external diaphragm piston area A.

In Figure 5, the brake is activated whenever the control voltage $U_{LS}$ is zero. In this situation, the brake perfectly counteracts the external force due to pressure, modelled through the equivalent depth voltage $U_{Z}$.

As seen in Figure 6, the reset signal r rises to 1 when both the actuator position module $\left|X\right|$ is greater or equal to half the stroke l and the control action U has the same sign as the actuator position. In this manner, when the limit switch is triggered, the actuator is free to move towards the center of the stroke but not in the opposite direction.

**Figure 7:**
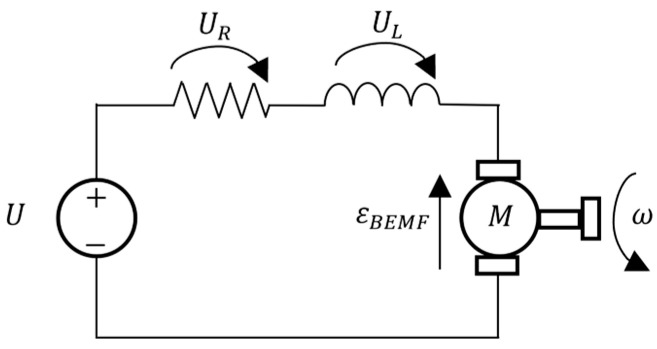
Generic DC motor model.

To estimate the energy consumed by the VBM, its instantaneous power $P_{VBM}$ must be integrated over time. The VBM power consumption is given by

(1) $$P_{VBM}=P_{0}+k_{d}\times \left|I\right|+U\times I$$

where $k_{d}$ is the driver electric loss coefficient, and $P_{0}$ is the power required to keep the various prototype electronics running: the Arduino, sensors, and driver. To find the current I, the generic model for a DC motor was considered and is presented in Figure 7.

From Kirchhoff’s second law applied to the loop,

(2) $$U=U_{R}+U_{L}+\varepsilon_{BEMF}⟺U=R\times I+L\times \frac{dI}{dt}+k_{BEMF}\times \omega$$

where R is the motor electric resistance, L is the electric inductance, and $k_{BEMF}$ is the back electromotive force constant. In the case of a linear drive, the angular velocity ω can be replaced with the linear velocity $\dot{x}$ and $k_{BEMF}$ is replaced with $k_{e}$, accounting for the linear drive pitch. As such, in steady-state conditions, the voltage applied to the linear drive can be given by

(3) $$U=R\times I+k_{e}\times \dot{x}$$

Solving for the current I,

(4) $$I=\frac{1}{R}\times \left(U-k_{e}\times \dot{x}\right)$$

**Figure 8:**
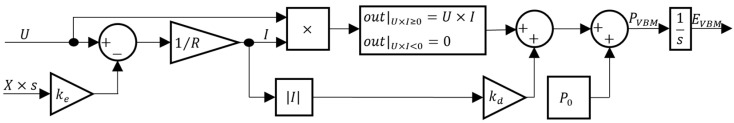
VBM energy estimation model.

Considering Equations (1) and (4), the energy estimation model is presented in the block diagram of Figure 8. Note that since there is no regenerative breaking when the actuator decelerates, the energy is not stored and as such, the instantaneous power must always be equal or greater than zero.

### 2.4. PM Model

**Figure 9:**
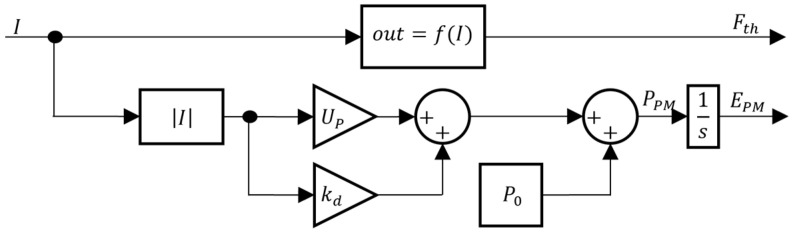
PM model.

The PM model was determined from the experimental values provided by the manufacturer in [20]. This experimental data will be presented in Section 2.5.3. In the experiment, the propeller was put in a water tank and its Bollard thrust was measured while recording the steady-state value of current drawn from the power supply set to a constant voltage. The PM model is presented in the block diagram of Figure 9.

In Figure 9, $U_{p}$ is the propeller driver supply voltage. The PM energy consumption is determined in a similar way to the VBM. It was assumed that the voltage $U_{p}$ supplied to the PM is constant and equal to the one used in the manufacturer experimental data.

### 2.5. Model Parameters

#### 2.5.1. Vertical Motion Model Parameters

As previously presented, the steady-state gain $K_{2}$ and time constant $T_{2}$ required to simulate the block diagram of Figure 3 were retrieved from [19]. Table 1 lists their values. For the purposes of this work, the prototype structure was considered nondeformable and thus, in the simulations, ψ=0.

#### 2.5.2. VBM Model Parameters

As stated in Section 2.3, the parameters of the linear actuator transfer function $K_{1}$ and $T_{1}$ (see Figure 4) were previously determined by the authors. However, in [14], the energy consumption of the prototype was not considered, so the parameters from Equations (1) and (4) must be determined. To that end, a simple experiment was devised in which the VBM actuator was powered on land with different voltage steps, (±2.5, ±5, ±7.5, ±10) V, and the piston position and power consumption were recorded to a VSC file on the memory card attached to the Arduino on the MCU. In the experiment, the actuator started at x=−20 mm with a zero-voltage set. Then, a positive voltage was applied until a total travel of approximately 40 mm was reached, at which point the voltage was then set to zero for approximately 5 s. Then, the symmetric voltage was applied until the actuator returned to the initial position, at which point the voltage was set back to zero. This process was repeated for each voltage step. The piston velocity was estimated using centered finite differences applied to the position measurements and the power was measured using the sensor described in Section 2. The piston velocity and power values considered were the average ones obtained for each voltage step. In this process, the value for $P_{0}$ was obtained using the average power measurements when the piston is still. In Equation (1), the current I is proportional to the linear drive motor torque, which depends on the external forces, the linear drive friction, and inertia. Since the experiment was conducted at ambient pressure, there are no external forces acting on the actuator. Due to the linear drive ball screw, friction is expected to be minimal and there are no inertial effects in steady-state conditions. In these conditions, Equation (1) can be rewritten as

(5) $$P_{VBM}=I_{nl}\times U+\left(P_{0}+k_{d}\times I_{nl}\right)$$

The current $I_{nl}$ is the linear drive no-load current. By using the data gathered from the experiment, plotting $P_{VBM}$ vs. U, and fitting Equation (5) to the data, the values for $I_{nl}$ and $k_{d}$ were found, allowing the parameters of Equation (1), $P_{0}$ and $k_{d}$, to be fully identified.

To find the current I of Equation (4), the value of R and $k_{e}$ are required. R was obtained by taking several measurements of the resistance at the motor terminals using a multimeter in different actuator positions. To determine $k_{e}$, the subsequent procedure was followed. Considering the conditions of the conducted experiment, Equation (4) can be rewritten as

(6) $$\dot{x}=\frac{1}{k_{e}}\times U-\frac{I_{nl}\times R}{k_{e}}$$

Using the experimental data retrieved in the experiment described above and the values of R and $I_{nl}$ previously determined, an $\dot{x}$ vs. U plot was generated. By fitting linear expression (6) to the data, the value for $k_{e}$ was obtained.

Finally, the value of the ratio $k_{z}/k_{u}$ is required to simulate the block diagram of Figure 4. As defined in [19], for an electromechanical solution,

(7) $$k_{z}=\rho \times g\times A\times \frac{\alpha }{2\times \pi \times \eta }$$

where α and η are the linear actuator transmission pitch and efficiency, respectively. As also defined in [19],

(8) $$k_{u}=\frac{k_{T}}{R}$$

where $k_{T}$ is the current-to-torque gain. On the other hand,

(9) $$T=k_{T}\times I\to \frac{dT}{dI}=k_{T}$$

Hence, for a linear drive,

(10) $$k_{T}=\frac{\alpha }{2\times \pi \times \eta }\times \frac{dF}{dI}$$

From [13], force and current can be related by

(11) $$\frac{dF}{dI}=\frac{1}{m_{I/F}}$$

where $m_{I/F}$ is the current/force slope on the manufacturer curves. Combining Expressions (8), (10) and (11),

(12) $$k_{u}=\frac{1}{R}\times \frac{\alpha }{2\times \pi \times \eta }\times \frac{1}{m_{I/F}}$$

Dividing Expressions (7) by (12),

(13) $$\frac{k_{z}}{k_{u}}=\rho \times g\times A\times R\times m_{I/F}$$

The VBM model parameters are presented in Table 2.

#### 2.5.3. PM Model Parameters

**Figure 10:**
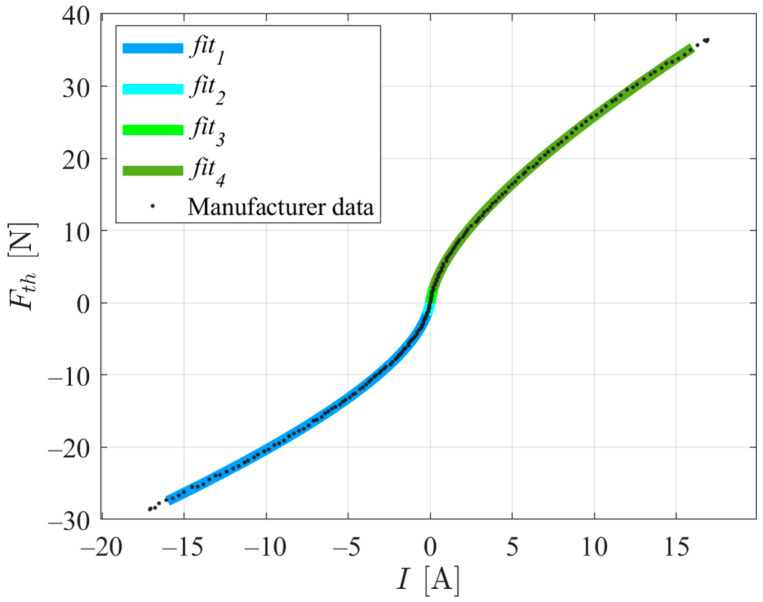
Bollard thrust data for the propeller and polynomial fits.

As stated in Section 2.4, the PM model was obtained using the Bollard thrust values experimentally determined by the manufacturer in [20]. These values are presented in the dotted curve of Figure 10. From the thrust and current drawn values for a 12 V power supply, for each rotation direction, a second-order polynomial was fitted using the least-squares method and forcing an origin interception. Figure 10 presents the experimental data provided by the manufacturer and four polynomial fits, ${fit}_{i}$, i=1…4, where ${fit}_{i}$ is represented by Equation (14) and a smooth transition between ${fit}_{1}$ and ${fit}_{2}$ and between ${fit}_{3}$ and ${fit}_{4}$ is ensured:

(14) $${fit}_{i}=a_{i}\times I^{0.5}+b_{i}\times I+c_{i}$$

**Figure 11:**
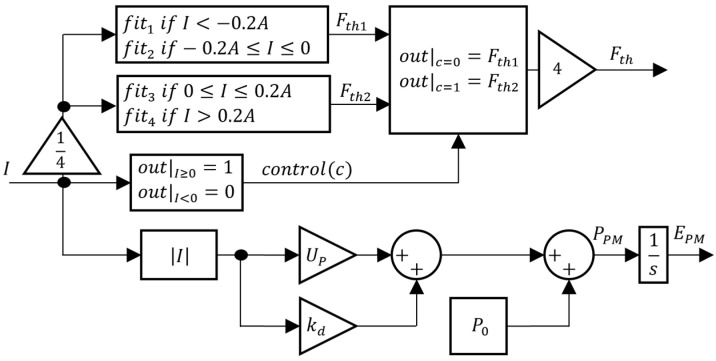
Updated PM model.

Considering the polynomial fits, the PM model from Figure 9 can be updated to the block diagram of Figure 11, where $F_{th1}$ and $F_{th2}$ are the negative and positive propeller thrust forces, respectively. When the current I is positive, the control signal c rises to 1 and the resulting thrust $F_{th}=F_{th2}$; otherwise, $F_{th}=F_{th1}$. It should be underlined that the data presented in Figure 10 are the characteristic curve for each propeller individually. Since the prototype has four propellers, the controller’s current demand is distributed evenly among them, with one fourth allocated to each. The overall force is thus four times the force provided by each propeller, as detailed in Figure 11.

The polynomial coefficients of each ${fit}_{i}$ are presented in Table 3.

## 3. Controllers

**Figure 12:**
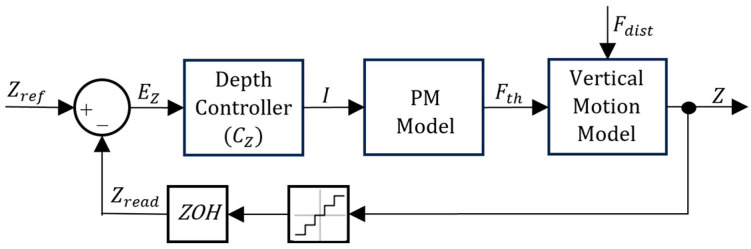
PM control architecture.

**Figure 13:**
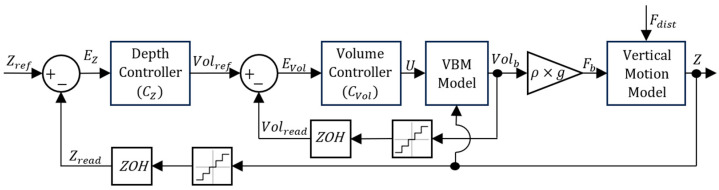
VBM control architecture.

To control the depth of the simulated prototype, different control architectures were devised for the PM and VBM. The PM only requires a standard architecture, with a single depth controller (please check Figure 12), because there is a static unequivocal relation between the current I and the force on the vertical motion model input $F_{th}$. This is not the case with the VBM, since there is a second-order, type 1 dynamic relation between the voltage applied to the VBM and the corresponding volume obtained. For this reason, an inner control loop was devised to control the VBM output (please check Figure 13). Since one of the main goals of the present work is to study the energy efficiency of both the VBM and PM, deadbands were implemented in each controller.

Regarding both Figure 12 and Figure 13, notice that each feedback branch has a Zero-Order Hold (ZOH) to model the signal acquisition between Arduino loops and a Quantizer block, accounting for sensor acquisition resolution. $E_{Z}$ is the depth error between the reference depth $Z_{ref}$ and the depth signal $Z_{read}$ calculated using the pressure read by the pressure sensor. In each control architecture, there is a depth controller $C_{Z}$. The output of $C_{Z}$ for the PM model is the current I and the output of $C_{Z}$ for the VBM controller is the reference volume ${Vol}_{ref}$. The input for $C_{Vol}$ is $E_{Vol}$, the volume error between ${Vol}_{ref}$ and the volume signal read by the position sensor ${Vol}_{read}$. The output from $C_{Vol}$ is the control action U for the VBM.

**Figure 14:**
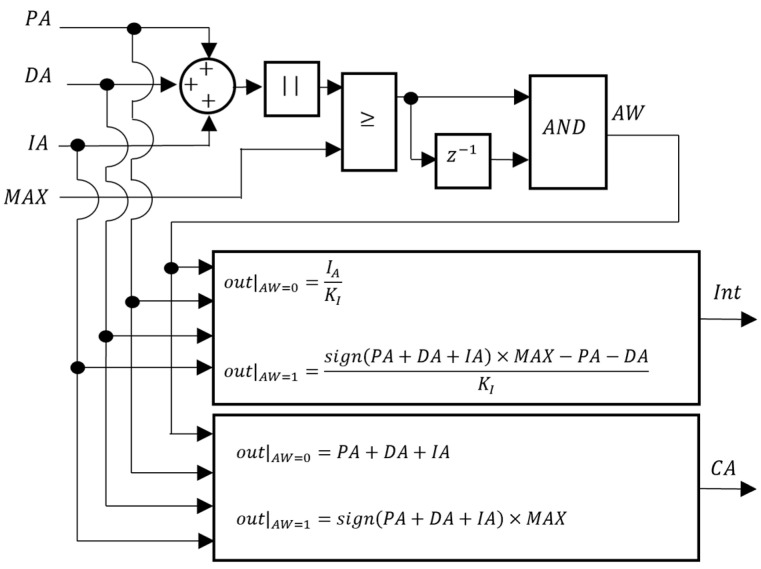
The block diagram of the anti-windup block.

To counteract possible disturbances acting on the prototype, each controller requires an integral action. To avoid integrator windup, an anti-windup scheme was implemented as presented in Figure 14. In this figure, PA, DA, and IA are the proportional, derivative, and integral control actions, respectively; MAX is the maximum value that the control action CA can take; and Int is the integral of the error. Whenever the control value equals or exceeds MAX for two consecutive time instants (AW=1), the value of CA is limited to MAX and the value of Int is calculated so that CA is also MAX. Integrator windup is thus prevented.

**Figure 15:**
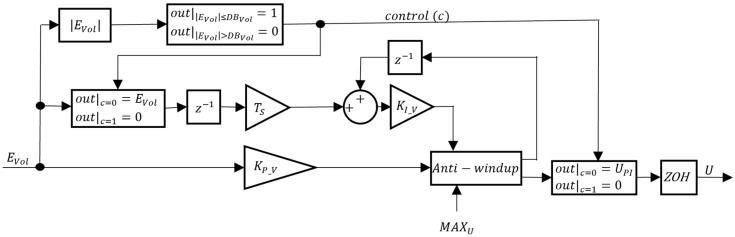
Block diagram of $C_{Vol}$.

$C_{Vol}$ was chosen to be a PI controller. The block diagram for $C_{Vol}$ is presented in Figure 15.

In Figure 15, $K_{I\_V}$ and $K_{P\_V}$ are the $C_{Vol}$ integral and proportional gains, respectively, and $T_{S}$ is the sampling time. To save energy, a volume deadband ${DB}_{Vol}$ is included, such that when the volume error $E_{Vol}$ is smaller than ${DB}_{Vol}$, the control signal c rises to 1 and the VBM is switched off. When this happens, the integrator input is set to zero to freeze the integrator. The PI controller action $U_{PI}$ is saturated to avoid exceeding the linear actuator input voltage range. Also, the ZOH block was added to simulate the holding of the control action U calculated value between Arduino sampling instants.

**Figure 16:**
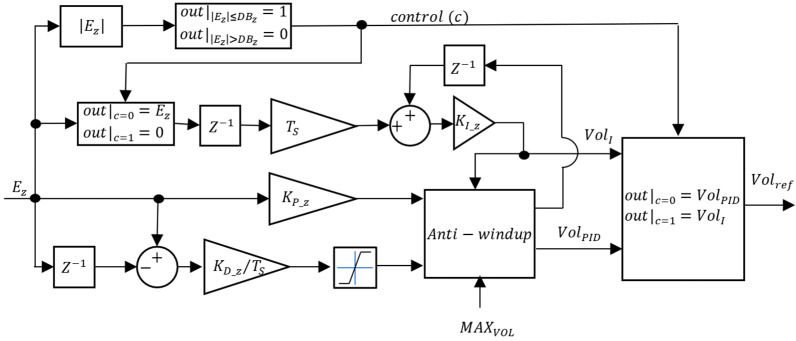
VBM depth controller ($C_{Z}$ = PID).

**Figure 17:**
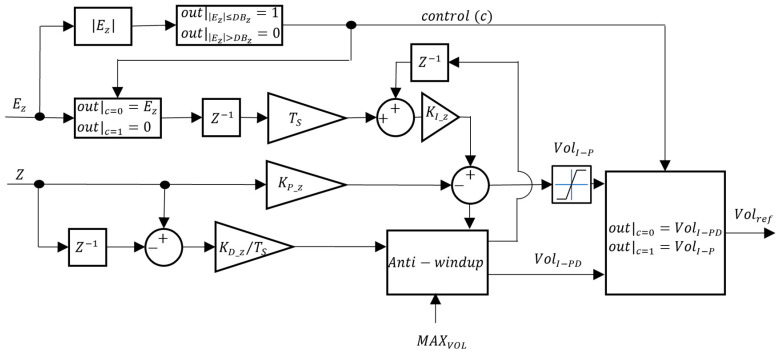
VBM depth controller ($C_{Z}$ = I-PD).

Regarding $C_{Z}$, PID and I-PD controllers were tested. The block diagrams for the VBM $C_{Z}$ controllers are presented in Figure 16 and Figure 17.

In Figure 16 and Figure 17, $K_{I\_Z}$, $K_{P\_Z}$, and $K_{D\_Z}$ are the integral, proportional, and derivative gains, respectively. A depth deadband ${DB}_{Z}$ was implemented in the VBM $C_{Z}$: when the absolute value of the depth error $E_{Z}$ is smaller than ${DB}_{Z}$, the control signal c rises to 1 and the integrator is frozen. Unlike the $C_{Vol}$, in this situation, the controller output is not zero but rather ${Vol}_{I}$ in the PID or ${Vol}_{I-P}$ in the I-PD. This ensures that if there is a constant disturbance, the integral part that counteracts it is still affecting the $C_{Vol}$ input. To limit the maximum value of the derivative action in Figure 16, due to possible excessive values whenever, for example, there is a step reference input, its value is limited to ${MAX}_{VOL}$.

**Figure 18:**
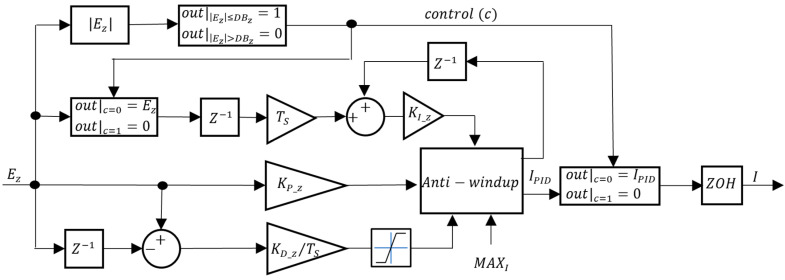
PM depth controller ($C_{Z}$ = PID).

**Figure 19:**
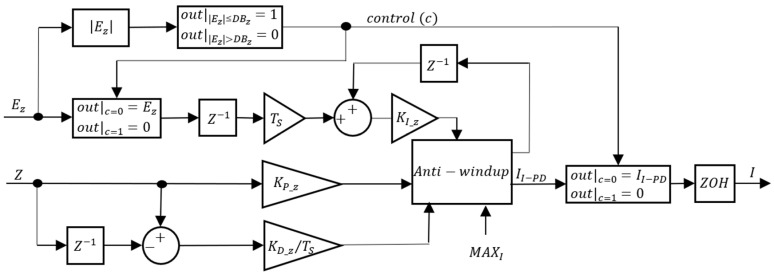
PM depth controller ($C_{Z}$ = I-PD).

The PM $C_{Z}$ controllers are similar to the VBM depth controllers with some adjustments due to the differences in control architecture. The block diagrams for the PM $C_{Z}$ controllers are presented in Figure 18 and Figure 19.

As seen in Figure 18 and Figure 19, a ${DB}_{Z}$ was also implemented in the PM depth controller, inside which the integrator is frozen. Since, in this case, the $C_{Z}$ output is the PM model input, when inside the deadband, the PM is switched off to save power. There is an anti-windup scheme to avoid saturation and integrator windup and the maximum value of the derivative action in Figure 18 is limited to ${MAX}_{I}$. The calculated value is again held with a ZOH to simulate the discrete time Arduino behavior. Table 4 lists the 12 different control structures tested.

## 4. Simulation Results

In this section, the models developed in Section 2 are simulated with the controllers presented in Section 3. Each control structure presented in Table 4 was simulated with two sets of controller tunings: (a) to increase performance and (b) to reduce energy consumption. These sets of parameters were obtained in a trial-and-error procedure, after intensive simulation runs. For $C_{Vol}$, the same gain values were used for every structure, $K_{P\_V}$ = 1 × 10^5^ V × m^−3^ and $K_{I\_V}$ = 1 × 10^5^ V × m^−3^ × s^−1^. The VBM gains for $C_{Z}$ are presented in Table 5 and for the PM in Table 6. Regarding the deadbands, the values used were ${DB}_{Z}$ = 0.1 m and ${DB}_{Vol}$ = 3.5 × 10^−5^ m^3^.

**Figure 20:**
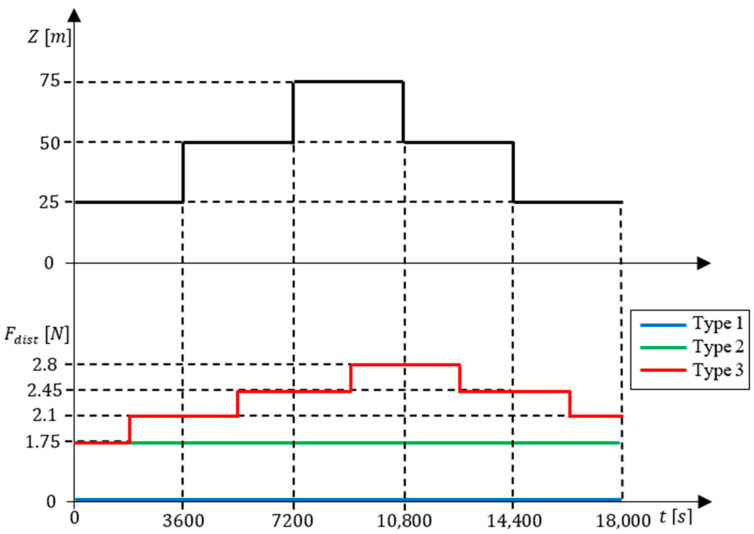
Depth reference signal (upper graph) and disturbances for each test type (lower graph).

The simulation trials were conducted in Matlab Simulink with a reference signal comprising several equal amplitude steps. Three types of tests were conducted: (1) without disturbances; (2) with a constant buoyancy disturbance throughout the entire test; and (3) with disturbances at middle points of each reference step in addition to the constant disturbance used in type 2 tests. Disturbances in type 2 tests simulated a neutral buoyancy trimming error, while those in type 3 tests simulated loading or unloading weights, or sudden density changes, as encountered in estuaries. The reference signal and the disturbances are depicted in Figure 20. Disturbances in type 3 tests are increasing steps of 35 cc up to 12,600 s and decreasing thereafter.

The energy consumption results for the prototype controlled by each structure and tuning are presented in Table 7. Results indicate that the PM requires less energy than any VBM control strategy, irrespective of the control structure used, in tests without disturbances (type 1 tests). However, when disturbances are present, as in type 2 and 3 tests, the energy consumption of the PM solution increases considerably, no longer offering the lowest consumption solution. It is also noticeable that the control strategy used with the PM does not seem to significantly affect the overall energy consumption for each type of test. In contrast, in the VBM case, the energy consumption does not significantly increase with disturbances; in fact, in many cases, it decreases. Additionally, for both the PM and the VBM, whether the depth controller is a PID or an I-PD, the usage of a depth deadband (even number control structures) does not seem to reduce the energy consumption of the prototype. However, in the VBM case, using a volume deadband led to considerable energy savings as it contributes to reducing the number of control action switchings.

**Figure 21:**
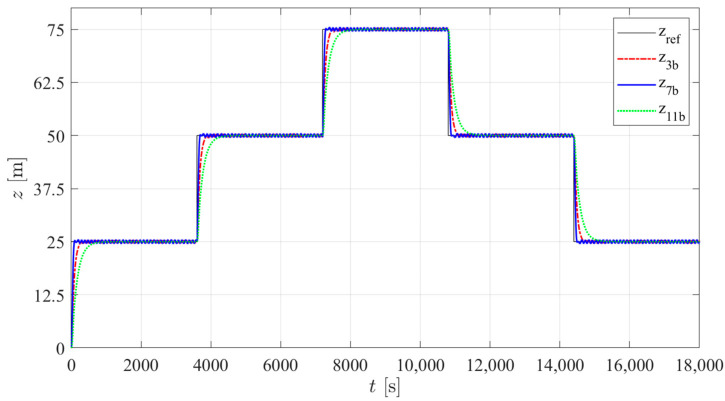
The simulated prototype depth for the selected control structures in type 1 trials.

**Figure 22:**
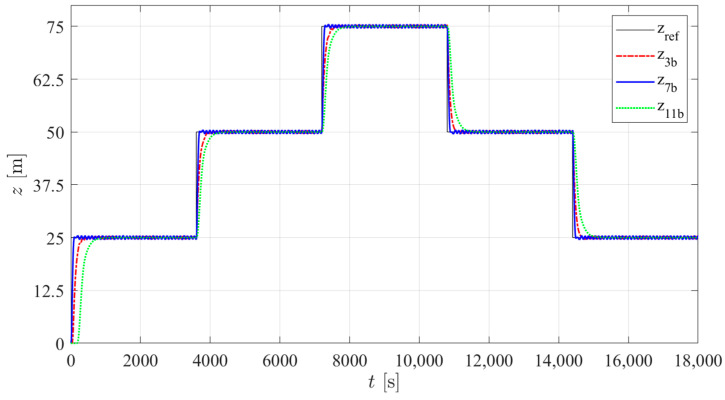
The simulated prototype depth for the selected control structures in type 2 trials.

**Figure 23:**
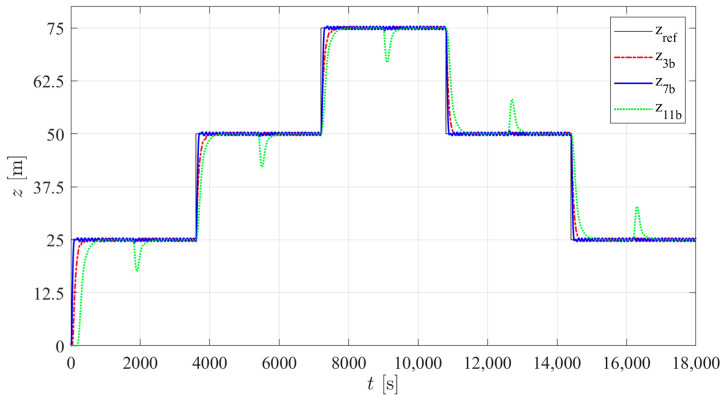
The simulated prototype depth for the selected control structures in type 3 trials.

According to the results presented in Table 7, three structures were selected with the lowest average energy consumption in the three types of tests. Among PID structures 1 to 4, tuning b of structure 3 was selected; among I-PD structures 5 to 8, tuning b of structure 7 was selected. As previously mentioned, no significant changes between the results of the different controller structures in PM were found, so among structures 9 to 12, only one controller (tuning b of structure 11) was selected. Figure 21, Figure 22 and Figure 23 show the simulated prototype depth during the three tests with the selected control structures. No significant overshoot is noticed in any of the responses and the VBM is faster than the PM. A delay in the first step is observed in type 2 and 3 trials. This is caused by the fact that for the first step, the integral part of the controller takes time to reach the value that will counteract the constant disturbance. In the case of controller 11b, this is particularly noticeable due to the low integral gain tuning required for low energy consumption.

**Figure 24:**
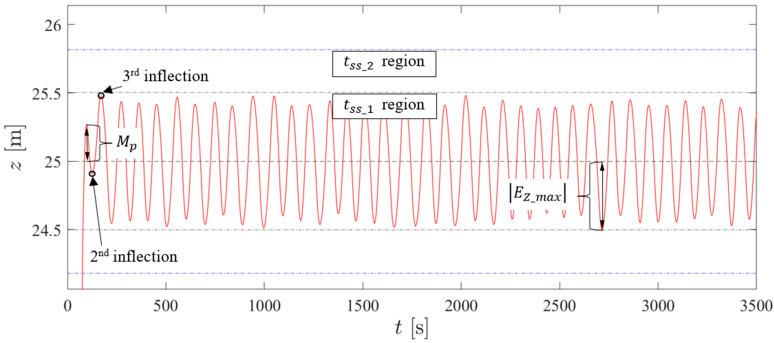
A graphical representation of the performance metrics: red curve is the depth trajectory, $t_{ss\_1}$ region is between grey dash-dot lines and $t_{ss\_2}$ region is between blue dash-dot lines.

To assess the performance of the selected control structures, conventional linear behavior metrics could not be used as the deadbands induce a nonlinear behavior. For this reason, several average performance metrics obtained in each test were adopted in this work: average overshoot $M_{p}$, average maximum error magnitude $\left|E_{Z\_max}\right|$, average settling times $t_{ss\_1}$ and $t_{ss\_2}$, and average disturbance error magnitude $\left|E_{Z\_dist}\right|$. Figure 24 presents a graphical representation of these performance metrics. The number of inflection points is counted after the system response crosses the target reference. $E_{Z\_max}$ is defined as the maximum error after the third inflection point of the vehicle for each new reference. $t_{ss\_1}$ is the time it takes for the prototype to reach the ±$E_{Z\_max}$ band of its own control structure, while $t_{ss\_2}$ is the time it takes for the vehicle to reach the ±$E_{Z\_max}$ band of the control structure that led to the highest $E_{Z\_max}$ in its respective test. $E_{Z\_dist}$ is the maximum error after a new disturbance. Table 8 presents the performance obtained for the previously selected controllers, as well as the energy consumption repeated from Table 7, for an easy assessment of the trade-offs between performance and energy consumption.

The results presented in Table 8 show that structure 7b is the fastest in every test by either settling time definition. The settling times obtained (between 80 and 130 s) are within the ones obtained for this type of vehicle [15]. Additionally, it leads to very little overshoot and presents the best results regarding robustness to the error caused by step disturbances. In fact, when a disturbance appears, structure 7b reacts very quickly and the maximum error after disturbance is only slightly bigger than the maximum error. Structure 11b leads to the smallest depth error but it is much slower than either 3b or 7b and has significantly less robustness to step disturbances, leading to a very large error. Overall, controller 7b, an I-PD controller with a deadband in the target volume, significantly outperforms the best PM controller regarding energy consumption, response time, and robustness to disturbances. The maximum steady-state error is around 0.5 m, which, although being significantly worse than the one obtained by the 11b PM controller (steady-state error below 0.02 m), is an acceptable value for hovering control, even for shallow waters, where depth control should be tighter [17].

## 5. Conclusions

This paper focused on the closed-loop depth control of a submersible platform, which can be used to monitor different ocean parameters through data collection from various sensors. Specifically, the energy consumption of two distinct actuation mechanisms for such platforms was examined: variable buoyancy and propeller actuated devices. Employing a prototype previously developed by the authors, this paper developed an intricate model of the platform utilizing both actuation solutions. Despite its linear foundation, the proposed model accommodates various nonlinearities such as saturations, sensor quantization, and actuator brake models. Additionally, it enables a straightforward estimation of the energy consumption associated with each actuation solution.

Several PID-based controllers were formulated and tested through simulation using the developed model. These controllers were employed to evaluate the dynamic response and energy demands of variable buoyancy and propeller actuated devices across diverse operational scenarios. The findings indicate that variable buoyancy systems can significantly reduce the energy required for hovering operations in the presence of buoyancy disturbances. In the scenarios analyzed in this study, adopting a variable buoyancy actuation system led to an energy consumption approximately 60% lower than that of an identical prototype powered by propellers. However, this reduction comes with a trade-off, as the variable buoyancy system exhibits a greater depth control error compared to the propeller-driven solution. Nonetheless, this compromise results in significantly faster settling times, with the variable buoyancy system outperforming the propeller-driven one.

Subsequent efforts will concentrate on refining control laws to potentially decrease the energy consumption of variable buoyancy systems. Specifically, the authors plan to explore the benefits of developing buoyancy disturbance observers toward this objective.

## Figures and Tables

**Table 1:** Vertical motion model parameters.

Parameter	Value	Unit
$K_{2}$	−7935.5	[ms^−1^m^−3^]
$T_{2}$	36.3	[s]

**Table 2:** Experimentally determined parameters.

Parameter	Value	Unit
$K_{1}$	4.44 × 10^−4^	[m×s^−1^×V^−1^]
$T_{1}$	9 × 10^−2^	[s]
A	7.7 × 10^−3^	[m^2^]
$k_{brake}$	1 × 10^2^	[V×s×m^−1^]
$P_{0}$	1.6	[W]
$I_{nl}$	7.884 × 10^−1^	[A]
$k_{d}$	1.427 × 10^−1^	[W×A^−1^]
R	4.370 × 10^−1^	[ohm]
$k_{e}$	2.051 × 10^3^	[V×s×m^−1^]
$k_{z}/k_{u}$	3.31 × 10^−2^	[V×m^−1^]

**Table 3:** PM model parameters.

i	$a_{i}$ [N×A^−1/2^]	$b_{i}$ [N×A^−1^]	$c_{i}$ [N]
1	−5.303	0.444	0.874
2	−1.603	4.493	0
3	1.349	6.950	0
4	6.124	0.737	−0.930

**Table 4:** Prototype control structures.

Actuating Device	$C_{Z}$	$C_{Vol}$	${DB}_{Z}$	${DB}_{Vol}$	Structure
VBM	PID	PI	No	No	1
			Yes	No	2
			No	Yes	3
			Yes	Yes	4
	I-PD	PI	No	No	5
			Yes	No	6
			No	Yes	7
			Yes	Yes	8
PM	PID	–	No	–	9
			Yes	–	10
	I-PD	–	No	–	11
			Yes	–	12

**Table 5:** Depth controller gains for the VBM.

Structure	Tuning	$C_{Z}=PID$		
		$K_{P\_Z}\left[m^{3}\times m^{-1}\right]$	$K_{I\_Z}\left[m^{3}\times m^{-1}\times s^{-1}\right]$	$K_{D\_Z}\left[m^{3}\times m^{-1}\times s\right]$
1–4	a	−1.8 × 10^−4^	−2 × 10^−6^	−2 × 10^−3^
	b	−9 × 10^−5^	−1 × 10^−6^	−1 × 10^−3^
		$C_{Z}=I-PD$		
5–8	a	2.5 × 10^−4^	−2 × 10^−6^	9 × 10^−3^
	b	7.5 × 10^−5^	−2 × 10^−6^	1.1 × 10^−3^

**Table 6:** Depth controller gains for the PM.

Structure	Tuning	$C_{Z}=PID$		
		$K_{P\_Z}\left[A\times m^{-1}\right]$	$K_{I\_Z}\left[A\times m^{-1}\times s^{-1}\right]$	$K_{D\_Z}\left[A\times m^{-1}\times s\right]$
9, 10	a	−5 × 10^−1^	−3 × 10^−3^	−5
	b	−1.2 × 10^−2^	−1 × 10^−4^	−3 × 10^−1^
		$C_{Z}=I-PD$		
11, 12	a	2 × 10^−1^	−5 × 10^−3^	1.2
	b	5 × 10^−3^	−3 × 10^−5^	5 × 10^−2^

**Table 7:** Energy consumption results for the simulated trials (structures selected for further comparison are highlighted in bold).

Actuating Device	Structure/ Tuning	Energy [kJ]		
		Type 1	Type 2	Type 3
VBM	1a	147.2	146.6	146.2
	1b	145	144.7	144.6
	2a	170.2	169.3	167.6
	2b	146.4	146.1	146
	3a	52.9	51	49
	**3b**	**46.2**	**44.1**	**43.7**
	4a	105	102.8	89.5
	4b	68.1	64.6	64.1
	5a	171.9	172.3	172.4
	5b	145.7	145.6	145.4
	6a	210.5	200.8	186.9
	6b	147.9	147.8	147.6
	7a	70.2	71.3	68.9
	**7b**	**41.9**	**41.6**	**41.9**
	8a	176.9	152.7	136.5
	8b	54.7	50.3	54.3
PM	9a	31.5	59	74.3
	9b	29.3	57.8	73.1
	10a	30.7	70.9	88.1
	10b	29.2	59.5	74.8
	11a	29.2	57.6	72.8
	**11b**	**28.8**	**57.4**	**72.6**
	12a	29	66.6	82.6
	12b	28.8	58	73.6

**Table 8:** Performance metrics for the selected structures.

	Type 1 Trials					
Structure	$M_{p}[\%]$	$\left|E_{Z\_max}\right|[m]$	$t_{ss\_1}[s]$	$t_{ss\_2}[s]$	$\left|E_{Z\_dist}\right|[m]$	Energy [kJ]
3b	0.6	0.39	395.2	343.7	−	46.2
7b	1.1	0.51	78.7	78.7	−	41.9
11b	0	0.001	1616	610.7	−	28.8
	**Type 2 trials**					
3b	1	0.38	413.5	318.6	−	44.1
7b	1	0.51	148.9	148.9	−	41.6
11b	0	0	2540.7	563.5	−	57.4
	**Type 3 trials**					
3b	1.2	0.38	482.4	316.1	0.675	43.7
7b	1	0.49	127.4	127.4	0.716	41.9
11b	0	0	1795.5	556.1	7.91	72.6

## Data Availability

Data are contained within the article.
